# Postnatal cerebral hemodynamics in infants with severe congenital heart disease: a scoping review

**DOI:** 10.1038/s41390-023-02543-z

**Published:** 2023-03-21

**Authors:** Alexandra Angela De Silvestro, Christian Johannes Kellenberger, Martina Gosteli, Ruth O’Gorman, Walter Knirsch

**Affiliations:** 1 Pediatric Cardiology, Pediatric Heart Center, Department of Surgery, University Children’s Hospital Zurich, University of Zurich, Zurich, Switzerland; 2 Center for MR-Research, University Children’s Hospital Zurich, University of Zurich, Zurich, Switzerland; 3 Children’s Research Center, University Children’s Hospital Zurich, University of Zurich, Zurich, Switzerland; 4 Department of Diagnostic Imaging, University Children’s Hospital Zurich, University of Zurich, Zurich, Switzerland; 5 University Library, University of Zurich, Zurich, Switzerland

## Abstract

**Abstract:**

Patients with severe congenital heart disease (CHD) are at risk for impaired neurodevelopment. Cerebral blood supply may be diminished by congenital anomalies of cardiovascular anatomy and myocardial function. The aim of this scoping review was to summarize the current knowledge on cerebral hemodynamics in infants with severe CHD. A scoping review was performed. Five databases were searched for articles published from 01/1990 to 02/2022 containing information on cerebral hemodynamics assessed by neuroimaging methods in patients with severe CHD within their first year of life. A total of 1488 publications were identified, of which 26 were included. Half of the studies used Doppler ultrasound, and half used magnetic resonance imaging techniques. Studies focused on preoperative findings of cerebral hemodynamics, effects of surgical and conservative interventions, as well as on associations between cerebral hemodynamics and brain morphology or neurodevelopment. Cerebral perfusion was most severely affected in patients with single ventricle and other cyanotic disease. Neuroimaging methods provide a large variety of information on cerebral hemodynamics. Nevertheless, small and heterogeneous cohorts complicate this field of research. Further studies are needed to improve our understanding of the link between CHD and altered cerebral hemodynamics to optimize neuroprotection strategies.

**Impact:**

- Postnatal cerebral hemodynamics are altered in infants with congenital heart disease (CHD) as compared to healthy controls, especially in most severe types such as single ventricle or other cyanotic CHD. Associations of these alterations with brain volume and maturation reveal their clinical relevance.
- Research in this area is limited due to the rarity and heterogeneity of diagnoses. Furthermore, longitudinal studies have rarely been conducted.
- Further effort is needed to better understand the deviation from physiological cerebral perfusion and its consequences in patients with CHD to optimize neuroprotection strategies.

## Introduction

Children with severe congenital heart disease (CHD) are at risk for delayed brain maturation, brain injuries, and clinical neurodevelopmental impairment.^1^ The underlying causes have not yet been fully established. Low intra- and perioperative cerebral oxygenation in CHD neonates has already been associated with structural and functional brain alterations.^2–5^ Moreover, anomalies in cardiac anatomy and function may impact brain development, causing alterations in cerebral hemodynamics and blood supply.

In healthy infants, postnatal cerebral perfusion increases rapidly within the first weeks of life,^6^ together with an increase of flow velocities in intracerebral vessels.^7^ This early increase is thought to reflect the closure of the arterial duct in neonates, as well as the high metabolic activity in the developing brain.^8,9^ Moreover, general body growth and weight gain lead to an increase in cardiac output within the first years of life and may also impact cerebral perfusion.^10,11^

The hemodynamic situation in infants with severe CHD differs from that in healthy peers and undergoes several changes within the first year of life. Prenatally, a brain-sparing mechanism has already been found in fetuses with single ventricle physiology.^12^ In contrary to healthy fetuses, vascular resistance in cerebral arteries was found to be decreased as compared to placental arteries, and therefore enhances blood flow to the brain.^12^ After birth, CHD patients may require prolonged systemic-to-pulmonary shunt (e.g., patent/stented arterial duct or surgical shunt) and cardiac surgery with the restructuring of vascular pathways and changes in cardiac function. Surgical treatment for severe CHD includes complete biventricular repair, such as arterial switch operation in d-transposition of great arteries (TGA) or staged palliation for patients with single ventricle CHD. Staged palliation consists of neonatal stage I procedure (i.e., Hybrid or Norwood), stage II procedure in infancy (bidirectional cavopulmonary anastomosis/Glenn procedure), and stage III procedure in early childhood (total cavopulmonary connection/Fontan procedure).

We hypothesized that brain development in patients with severe CHD will not only be influenced by neonatal cardiac surgery and perioperative management but also by the steady state of altered cardiac function and subsequent effects on cerebral blood and oxygen supply. Therefore, this work reviews postnatal cerebral hemodynamics in patients with severe CHD and its changes with surgical and conservative interventions. Insight into the cerebral hemodynamic situation can be achieved by neuroimaging techniques as they provide absolute values of cerebral blood flow and perfusion parameters.

This scoping review focuses on the postnatal course of cerebral hemodynamics in patients with severe CHD during early infancy, measured with neuroimaging methods. We aimed to (1) review neuroimaging methods for the measurement of cerebral hemodynamics and (2) summarize the current state of research on the postnatal course of cerebral hemodynamics in infants with severe CHD.

## Methods

This scoping review was carried out following the PRISMA guidelines.^13^ A research protocol was written in advance of the literature search (see Supplementary material).

### Eligibility criteria

Inclusion criteria were the following: (1) neonates and infants younger than 1 year of age, (2) severe CHD as defined by cardiac surgery required within the first 3 months of life, and (3) studies reporting absolute or relative values of cerebral hemodynamics, measured by imaging methods. Exclusion criteria were: (1) associated genetic syndrome, (2) prematurity, and (3) measurements performed intraoperatively or within 24 h postoperatively.

### Data search

A literature search was performed in collaboration with a librarian (MG) within Medline (OVID), EMBASE (via embase.com), Cochrane, Web of Science, and Scopus databases for the period between January 1990 and February 2022. The last data search update was conducted on February 11, 2022. The search string for Medline is provided in the Supplementary material; equivalent search strings were used in the further databases. Original, peer-reviewed research studies, including population-based register studies, retrospective and prospective cohort studies, cross-sectional studies, and case-control studies, were included. Reviews, editorials, or commentaries were screened for relevant studies but excluded from the results. No language limitation was performed for the data search.

### Screening

**Fig. 1:**
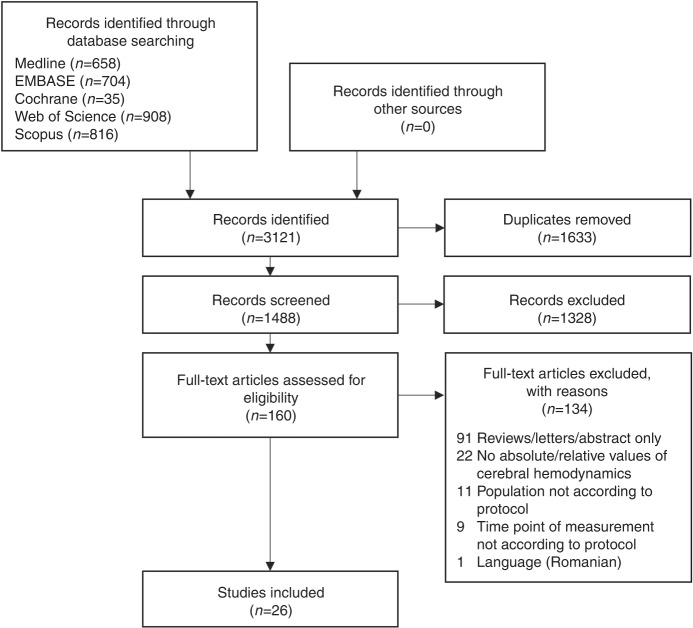
Flowchart for study selection.

In the first step, one investigator screened the title and abstract of each article on the basis of the eligibility criteria. In the second step, the full text of the screened studies was analyzed. A PRISMA flow diagram (Fig. 1) presents the screening process. The reasons for study exclusion were documented. Uncertainties about study eligibility were discussed with co-investigators (WK and RO), and eligibility was decided by consensus.

### Data charting process/data extraction

Studies were searched for: First author, year of publication, study design, country of study origin, sample size, study population and subgroups, control cohort, age of participants, imaging method and parameter, the time point of measurements, use of sedation, research focus, and results. Data items were selected from full-text publications.

## Results

### Description of the studies

A total of 160 studies were screened for eligibility by full text. Of those, 89 reviews, editorials, and commentaries were searched without the detection of additional publications. One study was excluded due to the Romanian language. Twenty-six studies were finally included in the review. Tables 1 and 2 present an overview of the characteristics and findings of dUS and MRI studies, respectively.

**Table 1:** Doppler ultrasound studies.

First author (Year)	Study population	Sample size	Research focus	Findings	Localization of measurement
**Single time point studies**					
Eckersley (2021)^31^	HLHS	31	Change of cerebral hemodynamics with birth	Postnatal MCA PI is higher in HLHS patients compared to controls. In HLHS patients, postnatal MCA PI is higher compared to fetal MCA PI (whereas in controls, postnatal PI decreases from 24 h onward and continues to stay lower as compared to fetal MCA PI). HLHS patients: MCA PI did not differ according to vasoactive support or positive pressure ventilation at any time point.	MCA (side unknown)
	Healthy controls	19			
Kussman (2007)^32^	HLHS/single ventricle	27	Difference of cerebral hemodynamics between patients with mBT shunt vs. RV-PA conduit, postop.	No differences in MCA velocities or RI between groups.	Right MCA
Day (1995)^28^	Diverse CHD	10	Effect of oxygen exposure on cerebral hemodynamics, preop.	No sign. change in MCA VTI with higher FiO_2_, but trend to decrease.	MCA (side unknown)
Toiyama (2010)^36^	Diverse CHD	8	Effect of hypoxic gas ventilation therapy on cerebral hemodynamics, preop.	Increase in MCA diastolic blood flow, sign. decrease of RI within 12 h after initiation of hypoxic gas ventilation therapy.	MCA (side unknown)
Li (2008)^47^	Diverse CHD	7	Effect of hypercarbia on cerebral hemodynamics, postop. after Norwood surgery with mBT shunt	PSV of MCA increased with hypercarbia increase of MCA PSV was greater between 40 and 50 mmHg PaCO_2_ than between 50 and 60 mmHg PaCO_2_.	MCA (side unknown)
Bianchi (2015)^39^	Diverse CHD	17	Effect of Milrinone (phosphodiesterase III inhibitor) on cerebral hemodynamics, preop.	PSV in ACA sign. increased at 24 h post initiation of Milrinone, trended in MCA. MV in ACA and MCA sign. increased over 24 h, remained sign. in ACA and trended in MCA at 48 h. No evidence for sign. change in EDV, RI, and PI of ACA or MCA with Milrinone.	ACA, MCA (side unknown)
Feria-Kaiser (2020)^48^	Diverse CHD	28	Association of preop. RI with postop. start of enteral feeding and spontaneous respiration	No difference in postop. start of enteral feeding and spontaneous respiration between patients with preop. low (<0.63) vs. normal (0.63–0.73) vs. high (>0.73) RI of BA.	BA
**Longitudinal studies**					
Saiki (2013)^30^	HLHS	9	Change of cerebral hemodynamics before/ after bilateral PAB (with prostaglandin infusion or ductal stenting)	No sign. postop. increase in cerebral artery velocities (PSV, EDV, MV). Some patients showed a postop. loss of diastolic flow.	Right MCA, proximal callosomarginal artery
Cheatham (2018)^27^	HLHS	18	Change of cerebral hemodynamics before/after hybrid stage I palliation	At 0, 2, 4, and 6 months: MCA PSV, EDV, and MV were lower in HLHS than in healthy controls (sign. at 6 months). Sign. higher MCA PI in HLHS at 2, 4, and 6 months compared to controls. Sign. increase of MCA PSV and MV over time in HLHS.	MCA (side unknown)
	Healthy controls	6			
			Correlation between dUS parameters and neurodevelopmental outcome	No sign. correlation of MCA dUS parameters with neurodevelopmental outcome at 6 months of age (Bayley III scales).	
Kim (2018)^29^	Diverse CHD	10	Change of cerebral hemodynamics before/after mBT shunt procedure	Preop. ICA PSV differed left and right, preop. PDV did not differ left and right. Preop.: 4 patients had bilateral reverse flow in ICA during diastole (ductal steal phenomenon). Postop. increase of bilateral ICA PSV, postop. increase of PDV at left ICA (contralateral to shunt), no change of PDV at right ICA (“relative decrease”). Postop. modified ICA RI was sign. higher at right side (unilateral to mBT shunt) than at left side. Postop.: one patient with bilateral diastolic reverse flow in ICA and bad outcome.	Bilateral ICA
Bertolizio (2015)^33^	Diverse CHD	24	Change of cerebral hemodynamics before/after Glenn procedure	From preop. to pre-discharge: decrease in MCA, MV, and PSV, no evidence for sign. change in peak end-diastolic velocity and RI. No evidence for sign. difference of MCA velocities between mBT shunt and RV-PA conduit group at any time point.	Right MCA
Jenks (2017)^43^	Diverse CHD	16	Difference in cerebral hemodynamics before/after surgery in patients undergoing SCP vs. non-SCP surgery	Preop. and before discharge: no evidence for sign. difference of cerebral artery RI’s between patient groups (patients with SCP vs. non-SCP surgery).	ACA, bilateral MCA
			Correlation of RI and neurodevelopmental outcome	No strong correlations of RI preop. and before discharge and neurodevelopmental outcome (Bayley III scales) at 12 months after surgery. Negative correlation of preop. RI and motor outcome in Vineland II scales.	
Ozturk (2021)^49^	Not defined (patients undergoing aortic arch surgery)	16	Change of cerebral hemodynamics before/after aortic arch surgery	Postop. sign. decrease in MCA RI, no evidence for sign. difference in PSV, EDV, MV, and PI.	MCA (side unknown)

*ACA* anterior cerebral artery, *BA* basilar artery, *CBF* cerebral blood flow, *CHD* congenital heart disease, *dUS* Doppler ultrasound, *EDV* end-diastolic velocity, *FiO*_*2*_ fraction of inspired oxygen, *HLHS* hypoplastic left heart syndrome, *ICA* A. carotis interna, *mBT shunt* modified Blalock–Taussig shunt, *MCA* middle cerebral artery, *MV* mean velocity, *PAB* pulmonary artery banding, *PaCO*_*2*_ arterial partial pressure of carbon dioxide, *PDV* peak-diastolic velocity, *PI* pulsatility index, *postop.* postoperative, *preop.* preoperative, *PSV* peak-systolic velocity, *RI* resistance index, *RV-PA conduit* right ventricle-to-pulmonary artery conduit, *sign.* significant, *SCP* selective cerebral perfusion, *VTI* velocity time integral.

**Table 2:** Magnetic resonance imaging studies.

First author (Year)	Study population	Sample size	Time point	Research focus	Findings	Method
**Single time point studies**						
Licht (2004)^24^	Diverse CHD	23	Preop. first surgery	iCBF assessment	Periventricular leukomalacia (in 28% of patients) was associated with decreased iCBF. No evidence for sign. difference in iCBF for different CHD types. Baseline iCBF was inversely and linearly associated with hemoglobin concentration.	PASL
				Effect of hypercarbia on iCBF	Periventricular leukomalacia was associated with poor iCBF reactivity under hypercarbia.	
					No evidence for sign. difference in CO_2_ reactivity for different CHD types.	
Wang (2006)^18^	Diverse CHD	3	Preop. first surgery	Effect of hypercarbia on iCBF	Increase of iCBF with hypercarbia in CHD neonates.	PASL, pCASL
	Healthy controls	1				
Durduran (2010)^37^	HLHS, TGA	12	Preop. first surgery	Effect of hypercarbia on iCBF	Increase of iCBF with hypercarbia.	PASL
Jain (2014)^22^	Diverse CHD	32	Preop. first surgery	Effect of hypercarbia on iCBF and iCMRO_2_	Increase in iCBF and decrease in oxygen extraction fraction with hypercarbia. No change in iCMRO_2_ with hypercarbia.	PC
Nagaraj (2015)^19^	Diverse CHD	43	Preop. first surgery	Global and regional iCBF in CHD subgroups vs. healthy controls	Average mean global and regional iCBF did not differ between CHD (all patients) and controls. Subgroup analyses: - single ventricle CHD have sign. lower iCBF than controls (global and regional in basal ganglia). No evidence for difference in global or regional iCBF between single ventricle and biventricular CHD. - cyanotic CHD have sign. lower regional iCBF in thalamus than controls. Cyanotic CHD have sign. lower iCBF in thalamus, basal ganglia and occipital white matter than non-cyanotic CHD. - aortic arch obstruction CHD’s have higher global and regional (basal ganglia, frontal and occipital white matter, thalami) iCBF than those without aortic arch obstruction. - CHD neonates ventilated on admission have sign. higher global and regional occipital white matter iCBF than non-ventilated CHD. - no relationship between gestational age at birth, lowest oxygen saturation, and SNAP score with CBF. - patients that had fetal cerebroplacental ratio <1 have sign. higher global and regional BG iCBF than those with cerebroplacental ratio >1. - CHD with antenatal increased isthmus flow have increased regional OWM iCBF than those without. - CHD with antenatal increased MCA RI have lower iCBF in OWM than controls.	pCASL
	Healthy controls	58				
Wintermark (2015)^20^	Atrioventricular canal defect	1	Preop. first surgery	Regional iCBF in CHD vs. control	iCBF in white matter was sign. increased in patient with atrioventricular canal defect vs. healthy controls. iCBF in cortical grey matter and basal ganglia did not differ sign. between atrioventricular canal defect patient and healthy controls.	PASL
	Healthy controls	3				
Fogel (2015)^38^	HLHS/single ventricle	34	Preop. stage II surgery	Correlation CBF and aortopulmonal shunt flow	Strong inverse correlation between CBF and APC/shunt flow on room air.	PC (cardiac)
				Effect of hypercarbia on CBF	Strong inverse correlation between CBF and APC/shunt flow with hypercarbia. This inverse relationship was stronger under hypercarbia than on room air. CBF as % of aortic flow increased under hypercarbia.	
Lim (2016)^21^	Diverse CHD	32	Preop. first surgery	CBF/iCBF and CDO_2_/iCDO_2_ in CHD vs. control	CBF and iCBF: no sign. difference in CHD vs. controls. CDO_2_ and iCDO_2_ in CHD is sign. lower than in controls. Age-dependent increase of CBF and iCBF is not sign. different btw CHD and controls. Age-dependent increase of CDO_2_ and iCDO_2_ is sign. different btw CHD and controls. Subgroup analysis: sign. lower CDO_2_ in single ventricle and TGA than in controls/no s. difference CoA and controls). Sign. correlation CDO_2_/iCDO_2_ and total maturation score. Sign. correlation CDO_2_ and total brain volume. No association between white matter changes and CBF or CDO_2_.	PC
	Healthy controls	31				
Fogel (2017)^42^	Diverse CHD	63	Preop. stage II surgery	Correlation of iCBF and cerebral oxygen delivery with brain lesions	CBF indexed to aortic flow (PC), CBF indexed to brain volume (PC) and iCBF (ASL) were not associated with brain lesions preop. to stage II. No sign. effect of cerebral oxygen delivery on brain abnormalities.	pCASL and PC (cardiac)
				Effect of hypercarbia on CBF	No sign. effect of CO_2_ reactivity on the odds of observing brain abnormalities.	
Kelly (2017)^26^	Diverse CHD	24	Preop. first surgery	Correlation of CDO_2_/iCDO_2_ and brain volumes/gyrification index	CDO_2_ correlates with brain volume (total and grey matter) and gyrification index. Indexing CDO_2_ per unit of brain volume weakened both the association with grey matter volume and gyrification index. Subgroup analysis: trend to lower iCBF and iCDO_2_ in CHD with left-sided and abnormal mixing lesions than in CHD with right-sided lesions. CDO_2_ sign. correlates with CBF but not with arterial saturation.	PC
Kelly (2019)^23^	Diverse CHD	39	Preop. first surgery	Correlation of CDO_2_ and microanatomical structure (diffusion-weighted imaging)	Linear relationship CDO_2_ and cortical orientation dispersion indices across many cortical regions. No evidence for sign. correlation of CBF or saturation alone with orientation dispersion indices. Absolute CBF for subgroups provided. Highest CBF in patients with pulmonary atresia, lowest CBF in patients with tricuspid atresia.	PC
Ng (2020)^25^	Diverse CHD	49	Preop. first surgery	Correlation of CDO_2_ and voxel-wise brain structure (tensor-based morphometry)	No sign. association between voxel-wise brain structure and CBF or CDO_2_. No sign. differences in CBF or CDO_2_ between cardiac subgroups (abnormal mixing vs. left-sided vs. right-sided lesions). Sign. positive correlation between CDO_2_ and total brain, cortical grey matter, and deep grey matter volumes.	PC
Bonthrone (2021)^41^	Diverse CHD	53	Preop. first surgery	Correlation of CDO_2_ and brain tissue atypicality indices	Sign. positive correlation of CDO_2_ with all brain tissue volume atypicality indices (total tissue volume, cortical grey matter, white matter, cerebellum, brainstem, left/right caudate, left/right lentiform nucleus, and left/right thalamus).	PC
					No evidence for sign. correlation of CDO_2_ with ventricle or extracerebral cerebrospinal fluid atypicality indices.	
				Correlation of CDO_2_ and neurodevelopmental outcome	No evidence for sign. correlation of CDO_2_ with neurodevelopmental outcome at 22 months (Bayley III).	
					Reduced CDO_2_ is indirectly associated with poor cognitive outcomes through the mediating effect of reduced volumetric brain development in several brain lesions.	

*APC/shunt* aortic-to-pulmonary collateral or aortic-to-pulmonary shunt, *ASL* arterial spin labeling, *CBF* cerebral blood flow, *CDO*_*2*_ cerebral oxygen delivery, *CHD* congenital heart disease, *CO*_*2*_ carbon dioxide, *DCS* diffuse correlation spectroscopy, *HLHS* hypoplastic left heart syndrome, *iCBF* indexed cerebral blood flow, *iCDO*_*2*_ indexed cerebral oxygen delivery, *iCMRO*_*2*_ cerebral metabolic rate of oxygen consumption, *MCA* middle cerebral artery, *PASL* pulsed ASL, *pCASL* pseudocontinuous ASL, *PC* phase contrast, *Preop.* preoperative, *sign.* significant, *TBM* tensor-based morphometry, *TGA* transposition of great arteries.

#### Study characteristics

The study design was prospective for 24 (92%) and retrospective for 2 (8%) studies. Twenty (77%) studies reported data for single time points: 16 studies assessed cerebral hemodynamics preoperatively, 2 studies postoperatively to the first cardiac surgery, and 2 studies preoperatively to stage II procedure in patients with single ventricle disease. Six (23%) studies had a longitudinal design and assessed changes before and after surgical procedures; all of them used dUS methods. Research foci varied widely and included the assessment of preoperative cerebral hemodynamics, the effects of surgical and conservative interventions, and the evaluation of associations between cerebral hemodynamics and brain morphology or neurodevelopment. Most of the included studies were published in English (25 studies, 96%), while one study was published in Spanish.

#### Population characteristics

In total, this scoping review includes the cerebral hemodynamic assessment of 629 patients. The mean CHD sample size was 24.2 (range 1–63). A control cohort was used in six (23%) studies.

Eighteen (69%) studies included different types of CHD with subgroup analyses in 11 studies, 7 (27%) studies focused on a single type of CHD, most frequently Hypoplastic Left Heart Syndrome (HLHS), and in one study, CHD diagnosis was not clearly defined. Use of sedation or general anesthesia was reported for six MRI studies, as well as for one dUS study. Eight (31%) studies did not report the sedation strategy during measurements.

### Methods of cerebral hemodynamic assessment

Cerebral hemodynamics were assessed by dUS (13 studies, 50%) and two MRI techniques (13 studies, 50%) using various hemodynamic parameters (Table 3).

**Table 3:** Hemodynamic parameters.

Abbreviation	Parameter	Formula	Unit	
**Doppler ultrasound**				
PSV	Peak-systolic velocity		cm/s	
EDV	End-diastolic velocity		cm/s	
MV	Mean velocity		cm/s	
RI	Resistance index	(PSV-EDV)/PSV		
PI	Pulsatility index	(PSV-EDV)/MV		
VTI	Velocity time integral		cm	
**Magnetic resonance imaging**				**Sequence used in studies**
CBF	Cerebral blood flow		ml/min	PC
iCBF	Indexed cerebral blood flow or ***cerebral perfusion***		ml/min/100 g or ml/min/100 ml^a^	ASL or PC
CDO_2_	Cerebral oxygen delivery	CDO_2_ = SaO_2_ × Hb × 1.36^b^ × CBF^21,23^	ml O_2_/min	PC
iCDO_2_	Indexed cerebral oxygen delivery	iCDO_2_ = SaO_2_ × Hb × 1.36^b^ × iCBF	ml O_2_/min/100 g	PC
O_2_D	Cerebral oxygen delivery	O_2_D = CBF × ((0.003 × PO_2_) + (1.34 × O_2_Sat × Hb))^42^	Not given	PC
iCMRO_2_	Cerebral metabolic rate of oxygen consumption	iCMRO_2_ = Ca × CBF × OEF^22^ (OEF: (SaO_2_−SvO_2_)/SaO_2_)^22^	ml O_2_/min/100 g	PC

*ASL* arterial spin labeling, *Ca* arterial oxygen concentration, *Hb* hemoglobin concentration, *MRI* magnetic resonance imaging, *OEF* oxygen extraction fraction, *O*_*2*_*Sat* oxygen saturation, *PC* phase contrast, *PO*_*2*_ partial pressure of oxygen, *SaO*_*2*_ arterial oxygen saturation, *SvO*_*2*_ venous oxygen saturation.

^a^Convertible to ml/min/100 g by multiplying with brain tissue density.

^b^The value 1.36 is the amount of oxygen bound per gram of Hb at 1 atmosphere (Hüfner’s constant).^21^

#### Doppler ultrasound

Cerebral vascular blood flow velocity is evaluated by dUS determining peak-systolic (PSV), end-diastolic (EDV), and mean velocity (MV) of the main cerebral arteries. These Doppler measurements, together with the assessment of velocity time integral (VTI), are angle-dependent. Nine studies assessed velocities, and one study VTI. Angle correction was not reported in the studies. The resistance index (RI) and/or pulsatility index (PI) was calculated in 10 dUS studies to investigate blood flow independent of the insonation angle. The middle cerebral artery was most often studied (MCA, *n* = 11), beside anterior cerebral (ACA, *n* = 2), proximal callosomarginal (*n* = 1), internal carotid (ICA, *n* = 1), and basilar artery (BA, *n* = 1). A disadvantage of dUS regarding the assessment of cerebral hemodynamics in neonates is the small vessel size; hence the caliber cannot be measured adequately, and absolute cerebral blood flow (CBF) calculation is not possible.^7,14^ If reported (*n* = 8), the pulsed-wave Doppler technique was used.

#### Magnetic resonance imaging: phase contrast and arterial spin labeling

Neonatal cerebral MRI is challenging due to the small brain size and high susceptibility to motion.^15^ Two noninvasive MRI techniques had been used in the included studies to estimate CBF without the application of contrast agents: phase contrast (PC) in eight studies and arterial spin labeling (ASL) sequences in six studies; one study combined both methods.

In PC MRI, CBF (ml/min) is calculated from the velocity and cross-sectional area of the blood within the main feeding arteries, usually measured in both ICAs and the BA.^16^ Blood velocity is estimated using bipolar flow-encoding gradients.^16^ Vascular flow can be converted to the average (whole-brain) indexed cerebral blood flow (iCBF, in ml/min/100 g or ml/min/100 ml brain tissue) by dividing the total vascular flow by the total brain volume (in grams or milliliters).^16^ The terms “indexed cerebral blood flow/iCBF” and “cerebral perfusion” are used as synonyms in this review.

In ASL, cerebral perfusion (in ml/min/100 g) is calculated from the brain tissue signal using a subtraction method.^16^ Radiofrequency pulses applied to the neck region invert the blood signal during a preparation phase.^16^ After a certain delay allowing the inverted spins in cervical arteries to reach the brain tissue, the labeled image is acquired and subtracted from a native image, resulting in an image with signal intensities proportional to CBF.^16^ The included studies used different ASL methods: three used pulsed ASL (PASL), two used pseudocontinuous ASL (pCASL), and one study combined both (Table 2). Whereas PASL uses a thick slab of 10 cm for the inversion pulse in a short time range of 5–20 ms, pCASL uses a thinner slab for a longer duration (1–2 s).^16^ pCASL is more dependent on blood flow velocity, but the quality of pCASL tends to be higher than for PASL images.^17^ For either technique, multiple control-label sets need to be acquired to increase the signal-to-noise ratio, requiring a longer acquisition time with the difficulty of possible motion artifacts.^16^ Spin labeling signal is furthermore dependent on patient characteristics like age and hematocrit, and measurements need to be adapted accordingly.^16,18^ In contrast to PC, ASL allows not only the assessment of global cerebral perfusion but also regional perfusion, as acquired in two^19,20^ of the six ASL studies. ASL has a shorter post-processing time than PC, whereas PC MRI has a higher signal-to-noise ratio.^16^

### Research topics

#### Preoperative cerebral hemodynamics

##### CHD vs. healthy neonates

After birth, a preoperative age-dependent increase of global cerebral perfusion was found in neonates with CHD, similar to the physiological findings in healthy controls.^21^ While preoperative global cerebral perfusion values did not differ between cohorts of diverse CHD patients and healthy controls in two studies (determined by PC^21^ and ASL,^19^), they were lower than in comparable healthy literature reports in one study (determined by PC^22^). In subanalyses of specific CHD types as compared to healthy controls, lower cerebral perfusion was revealed in patients with single ventricle disease, both globally and regionally in the basal ganglia and in the thalami in patients with cyanotic CHD.^19^

Similarly, an age-dependent increase of preoperative cerebral oxygen delivery (CDO_2_ and indexed per brain volume: iCDO_2_; calculated from CBF/iCBF, hemoglobin and oxygen saturation, Table 3) was found in a cohort of different types of CHD.^21^ But, in contrast, this age-dependent increase of CDO_2_/iCDO_2_ was weaker in CHD patients than in controls.^21^ In disease-specific subanalyses, CDO_2_ of patients with single ventricle disease and TGA was lower than in controls, whereas no difference was found between patients with coarctation of the aorta and controls^21^.

##### Differences within CHD subgroups

Patients with cyanotic CHD were shown to have similar global cerebral perfusion but decreased regional perfusion in thalami, basal ganglia, and occipital white matter, as compared to patients with acyanotic CHD.^19^ On the other hand, patients with aortic arch obstruction had increased global perfusion, as well as regionally in thalami, basal ganglia, occipital, and frontal white matter, than those without aortic arch obstruction.^19^ These findings are supported by Kelly et al.,^23^ who reported the lowest global blood flow (ml/min) and CDO_2_ values (ml O_2_/min) for HLHS, Truncus arteriosus communis, and tricuspid atresia, medium values for TGA patients and the highest values for aortic coarctation, pulmonary atresia, and Fallot patients, although those values were not indexed per brain volume.

No differences in cerebral perfusion/iCBF were found for the following comparisons: uni- vs. biventricular CHD,^19^ TGA with vs. without preoperative balloon-atrial septostomy^19^ within cardiac subgroups (not further specified).^24^ CBF of CHD groups with abnormal mixing vs. left-sided vs. right-sided lesions did not differ, either.^25^ No CDO_2_ difference between male and female patients was found.^26^

#### Therapeutic effects on cerebral hemodynamics

##### Duct- or surgical shunt-dependent pulmonary and systemic circulation

*Patent ductus arteriosus (PDA)*

In healthy infants, cerebral diastolic velocity increases within the first months after birth^27^ due to the closure of PDA and an increase in cardiac output.^10^ In several types of severe CHD, persistent blood flow across the arterial duct is needed either by using prostaglandin E1 infusion or by duct stenting. Duct-dependent systemic circulation may result in an unfavorable balance of systemic and pulmonary perfusion.^28^ Furthermore, in HLHS patients undergoing Hybrid palliation with pulmonary artery banding and patent ductus arteriosus, postoperative cerebral perfusion remains duct-dependent and retrograde through the aortic arch.^27^

In some patients, already preoperatively, not only low diastolic velocity but a diastolic runoff was detected in cerebral arteries and described as “ductal steal phenomenon”.^29^ It implies a relatively large pulmonary flow due to overshunting.^29^ In HLHS patients after Hybrid palliation, cerebral diastolic velocity remained low^27^ or even decreased.^30^ The low diastolic velocity causes the PI to remain high both pre-^27,31^ and postoperatively.^27^ Systolic velocities of cerebral arteries did not change from pre- to postoperative, up to 7 days after Hybrid palliation.^30^ In later follow-ups at 2, 4, and 6 months, an increase with time was found, as expected from infant growth.^27^ Within an observational period from birth to the age of 6 months before and after Hybrid palliation, all flow velocities (systolic, diastolic, mean) in cerebral arteries of HLHS patients with open ducts remained lower than in healthy controls.^27^

*Surgical shunts: Systemic-to-pulmonary shunt (modified Blalock–Taussig (mBT) shunt) and right ventricle-to-pulmonary artery (RV-PA) conduit*

The mBT shunt connects the right subclavian and right pulmonary artery, whereas the RV-PA conduit connects the right ventricle and pulmonary artery. Side-specific measurements of cerebral arterial velocities were conducted pre- and postoperative to mBT shunt procedure in a study including diverse types of CHD.^29^ Postoperatively, a lack of increase in diastolic velocity was shown unilateral to the shunt, right-sided, with a consequently higher RI,^29^ similar to the observations in patients with an open arterial duct. In all four patients with preoperative ductal steal phenomenon (reverse diastolic flow), no shunt steal was detected postoperatively to the mBT shunt procedure.^29^ Bilateral PSV and contralateral diastolic velocity of ICA increased after the mBT shunt procedure.^29^

The “ductal steal” or “shunt steal” phenomenon has the potential for cerebral hypoperfusion and contribution to subtle neurologic injury.^27,29^ In one patient, a shunt steal, manifested by early diastolic reverse flow at both ICAs after the mBT shunt procedure,^29^ was observed. The patient died 1 week after surgery.^29^

Comparing patients after the mBT shunt vs. RV-PA conduit procedure, no differences in cerebral blood velocities and RI of the right MCA were found.^32^

*From surgical shunt to cavopulmonary anastomosis in univentricular palliation*

With Glenn operation (stage II in univentricular heart palliation), the shunt (PDA, mBT, or RV-PA shunt) is removed, and a superior bidirectional cavopulmonary anastomosis is created. After shunt removal (mBT or RV-PA shunt), diastolic velocity or RI did not change postoperatively, but systolic and mean velocities decreased in comparison with preoperative values (right-sided measurements).^33^ Both pre- and postoperatively, mean and diastolic velocity remained lower and RI higher than reported for healthy infants.^33^ Consistent with the findings from postoperative to stage I,^32^ no differences in cerebral hemodynamic variables between patients with mBT shunt and RV-PA conduit were found pre- or postoperatively to Glenn surgery.^33^

##### Vasoactive agents

*Hypercarbia therapy*

Increasing partial pressure of carbon dioxide in arterial blood (PaCO_2_) causes cerebral vasodilation.^34^ Moreover, a decrease in partial pressure of oxygen (PaO_2_) with hypoxic gas ventilation therapy induces an increase in pulmonary vascular bed resistance with a consecutive decrease in pulmonary blood flow.^35^ These reactions lead to a reduction of diastolic runoff of cerebral to pulmonary circulation by PDA^36^ or other systemic-to-pulmonary shunts.

In the reviewed studies, hypercarbia resulted in an increase of diastolic velocity and a consequential decrease of RI in dUS measurements of MCA,^36^ as well as in an increase of global cerebral perfusion in MRI methods (ASL,^24,37^ PC^22,38^). In line with this finding, the opposite, higher fractions of inspired oxygen, resulted in a trend of decreased blood flow in MCA (VTI, measured by dUS).^28^ Measurements were done before cardiac surgery^22,24,28,36,37^ in studies including different types of CHD, as well as before Glenn surgery,^38^ suggesting that CO_2_ reactivity of cerebral vessels is competent at both time points. CO_2_ reactivity of cerebral perfusion did not differ between CHD types.^24^

Together with a preoperative increase of brain perfusion with hypercarbia, Jain et al.^22^ found an increase in venous O_2_ saturation in the superior sagittal sinus and a decrease in oxygen extraction fraction. The cerebral metabolic rate of oxygen consumption (CMRO_2_, Table 3) did not change with hypercarbia.^22^

*Milrinone*

Milrinone is a specific phosphodiesterase III inhibitor that increases cardiac output and decreases systemic vascular resistance, which leads to a redirection of pulmonary and systemic blood flow.

Bianchi et al.^39^ found an effect of Milrinone on cerebral hemodynamics (ACA and MCA) during short-term preoperative use in CHD neonates with duct-dependent perfusion. Similar to the increase of cardiac output (25% increase from baseline) over the 48 hours of therapy, cerebral artery velocities (PSV and MV) increased to a value of 30–40% above baseline. Both increases in cardiac output and cerebral artery velocities were greater than would be expected by term infant physiologic adaptation after birth.^39^ However, no effect of Milrinone^39^ or other vasoactive support^31^ was found on EDV, RI, and PI.

#### Associations of cerebral hemodynamics with brain morphology and function

CHD neonates are at risk for delayed brain maturation, reduced brain volumes, and brain lesions like white matter injuries or focal strokes.^1^ Furthermore, the neurodevelopmental outcome in later life may be impaired.^40^

##### Brain maturation and volume

Preoperative cerebral perfusion was not strongly associated with delayed brain maturation (incomplete closure of the operculum) or microcephaly.^24^ In contrast, preoperative cerebral oxygen delivery (CDO_2_ and iCDO_2_) was associated with multiple variables of brain maturation and volume. CDO_2_ was linked with total brain volume,^21,25,26^ grey matter volume^25,26^ and iCDO_2_ was associated with grey matter volume, too, but to a lesser extent than non-indexed CDO_2_.^26^ CDO_2_ and iCDO_2_ were both associated with total maturation score,^21^ and CDO_2_ with gyrification index (but not after indexing per total brain volume^26^).

In microstructural analyses, CDO_2_ was associated with cortical orientation dispersion index (ODI)^23^ and with atypicality indices within the whole brain tissue ^41^ as further parameters of brain maturation. Conversely, voxel-wise brain structure, investigating microstructural brain shape and volume, was not associated with CDO_2_ or CBF.^25^

##### Brain lesions

Preoperatively, periventricular leucomalacia (PVL) was associated with lower iCBF, as well as with poor CO_2_ reactivity.^24^ Another preoperative study found no relation of white matter changes with CBF/iCBF or CDO_2_/iCDO_2_,^21^ and investigation of univentricular patients preoperative to stage II surgery revealed no relation of brain lesions (e.g., focal tissue loss, PVL) and CBF/iCBF, O_2_ delivery (O_2_D), or CO_2_ reactivity.^42^

##### Neurodevelopmental outcome

No evidence for strong correlations of cerebral blood velocities or RI/PI measurements and neurodevelopmental outcome at 6 or 12 months was found in dUS studies^27,43^ at the predefined time range of this review. To date, no study assessed the association between cerebral perfusion and neurodevelopmental outcome. For CDO_2_, no direct relation was found with neurodevelopmental outcome at 22 months, but reduced CDO_2_ was indirectly associated with poor cognitive abilities in early childhood through the mediating effect of reduced volumetric brain development in several brain regions.^41^

## Discussion

Twenty-six neuroimaging studies investigating postnatal cerebral hemodynamics in patients with severe CHD were identified. A large variety of topics was investigated at different time points in the course of cardiac treatment within the first year of life, using dUS and MRI methods. Comparisons of cerebral hemodynamics with healthy controls, differences within CHD diagnoses, as well as therapeutic effects and associations with brain morphology and function were assessed.

### Imaging methods

dUS and MRI techniques as complementary methods are sensitive to different developmental and pathological changes in the brain. While MRI studies revealed knowledge of global and regional cerebral perfusion together with associations of brain morphology, dUS studies provided knowledge of cerebral vascular flow and resistance, even longitudinally, revealing hemodynamic changes with treatment. For delicate cohorts such as CHD patients, ultrasound is a noninvasive bedside tool with good accessibility. On the other hand, objectivity and reproducibility may be limited. In contrast, PC methods showed high interobserver agreement,^21^ and both PC and ASL results correlated well with measurements using diffuse optical and correlation spectroscopy.^22,37^ Near-infrared spectroscopy (NIRS) methods are additional techniques to provide noninvasive insight into microvascular tissue oxygenation. They evolved into important clinical tools offering bedside neuromonitoring and, furthermore, advanced calculation of oxygen metabolism and indices of microvascular CBF.^22^ However, because of spatial limitations (standard use at frontal cortex only, limited penetration depth) and technical challenges (challenges in separation of hemodynamic changes arising from cerebral or extracerebral tissues, lack of standardized signal processing and analysis methods),^44^ these methods were not within the scope of this review.

### Cerebral hemodynamics

The age-dependent increase of cerebral perfusion in the early life of healthy newborns had been described to represent high metabolic activity in the neonatal period, e.g., due to high energy consumption of oligodendrocytes for myelination.^6,45^ In CHD patients, the age-dependent increase of CBF/iCBF was comparable, but the increase of CDO_2_/iCDO_2_ was weaker, and Doppler flow velocity values remained lower as compared to those in healthy newborns.^21^

As a major finding, global and/or regional cerebral perfusion was decreased in patients with single ventricle disease and other cyanotic CHD.^19^ However, the cause is not yet entirely clear and may be multifactorial. The ductal- or shunt steal phenomenon with diastolic runoff into the lung may result in lower cerebral perfusion, as seen in the most severe CHD diagnoses. A number of dUS studies described this phenomenon,^27,29–31^ reporting decreased diastolic velocity and high RI and PI in cerebral arteries. In consequence, these findings imply that the brain-sparing mechanism seen in fetal patients with severe CHD, consisting of low cerebral resistance to enhance cerebral perfusion,^12^ seems not to persist after birth, possibly as a result of the hemodynamic shunt effect. The steal phenomenon may not only compromise blood flow to the brain but also to other vital organs,^31^ such as the heart (coronary arteries), kidneys, or the gastrointestinal tract, with a risk for corresponding hypoperfusion injuries. It seems like the hemodynamic balance could only be improved by the closure or removal of the systemic-to-pulmonary shunt. Nevertheless, MCA RI did not change after Glenn surgery (bidirectional cavopulmonary anastomosis and removal of shunt), RI remained higher, and velocities were lower than reported for healthy infants.^33^ As a further effect of Glenn surgery, central venous pressure increases, resulting in an impaired venous blood flow from the brain to the heart. However, an acute impact on cerebral hemodynamics has not been described in the analyzed studies. Two years after the Glenn surgery, a trend toward impaired neurodevelopmental outcomes associated with higher central venous pressure was reported.^46^

Concerning the type of systemic-to-pulmonary shunt, no difference in cerebral hemodynamics was detected between patients treated with mBT shunt vs. RV-PA conduit directly after shunt procedure,^32^ and both pre- and postoperative to Glenn surgery.^33^ A potential bias factor of these findings is the unilateral measurement of the right MCA only, as side-specific differences may not have been detected. Moreover, because of bilateral blood supply via the circle of Willis, MCA measurements may provide shunt effects to a lesser extent than ICA measurements.

In addition to the impacts of systemic-to-pulmonary shunts, cerebral perfusion is influenced by blood oxygenation. CO_2_ and O_2_ reactivity of the cerebral vessels in CHD patients are existent: cerebral perfusion increased with hypercarbia.^22,36–38^ But, cyanotic patients were found to have reduced regional thalamic perfusion, and single ventricle patients were found to have reduced global and regional perfusion as compared to healthy controls.^19^ Cerebral autoregulation to guarantee adequate blood supply may therefore be impaired or disrupted (e.g., by the shunt steal effect) in the most severe types of CHD. Unfortunately, blood pressure or intracranial pressure was not assessed in the reviewed studies to accurately investigate cerebral autoregulation.

Reduced CBF may be a critical insult.^31^ In CHD patients, low cerebral perfusion was associated with PVL.^24^ Other brain lesions like WMI^21^ were not associated and may represent rather acute events, e.g., during surgery. No association between cerebral perfusion and brain microstructure or maturation has been found so far. But, CDO_2_ as a combined parameter of perfusion, arterial oxygenation saturation, and hemoglobin concentration, was found to be decreased in CHD patients as compared to healthy controls,^21^ and was associated with brain maturation,^21,23,26,41^ as well as with cognitive abilities in early childhood through the mediating effect of reduced volumetric brain development.^41^ Therefore, low cerebral perfusion combined with hypoxemia, causing cerebral hypoxia and hindering optimal oxygen and nutrition supply, may contribute to the alterations in brain development in CHD patients.

### Limitations

The heterogeneity of cohorts with different types of CHD and surgical procedures is a well-known limitation of this research field. Sample sizes were rather small, and a number of publications reported results for the total cohort without taking differences in diagnoses into account. Most of the studies assessed a preoperative time point, and long-term studies are rare. Longitudinal MRI studies are lacking completely. One study^29^ found asymmetries in cerebral Doppler measurement, but most ultrasound studies investigated one side only, most frequently the right side, whereas other studies did not report the side at all. Furthermore, although associations of cerebral hemodynamic parameters with age, the existence of shunt, or the use of vasoactive agents during measurement have been found, most of the MRI studies did not use these variables as covariates in their analyses. It was shown that CDO_2_ is associated with brain volume ^21,25,26^ but not all studies adjusted for it.

As a further limitation of this review, we included neuroimaging data only. Data from other techniques as NIRS or amplitude-integrated electroencephalography (aEEG), may additionally contribute to the understanding of altered cerebral hemodynamics and its consequences.

### Research gap

The alterations in cerebral hemodynamics of CHD neonates need to be studied in more detail, as they may represent a cause for the deviations from healthy neurodevelopment, in addition to the higher frequency of acute brain damage caused by invasive treatment in early life. Long-term studies assessing cerebral perfusion after the postnatal time point are needed to investigate the chronic state of cerebral blood and oxygen supply in CHD patients. Furthermore, the effect on long-term neurodevelopmental outcomes (after 2 years of life) is of high clinical interest but has not yet been explored.

Objective measurement of cerebral hemodynamics is complicated by numerous patient-, therapy- or method-related influences. Larger sample sizes, for example, provided by multicenter studies, would be beneficial to study these factors more systematically. Furthermore, the association between cardiac function (e.g., by cardiac output) and cerebral hemodynamics has not been well investigated yet. In addition, more information on regional perfusion may uncover signs of redistribution or brain-sparing effects.

## Conclusion

To date, a large variety of cerebral hemodynamic assessments have been conducted in CHD infants. Cerebral perfusion of most severe cases like single ventricle and other cyanotic disease is decreased, and reduced cerebral oxygen delivery has been associated with delayed brain maturation in mixed CHD cohorts. The clinical impact on long-term neurodevelopment is not yet clear. Further studies researching the longitudinal course of cerebral hemodynamics in patients with CHD and its impact on neurodevelopmental outcomes are key for optimal care and neuroprotection.

## Supplementary information

Supplementary Materials

## Data Availability

All data generated or analyzed during the current study are available from the corresponding author upon reasonable request.
