# Supplementary material for: Postnatal cerebral hemodynamics in infants with severe congenital heart disease: a scoping review
Source: Pediatr Res. 2023 Mar 21;94(3):931–43. doi: 10.1038/s41390-023-02543-z (PMC10444615; doi:10.1038/s41390-023-02543-z)
Supplement: Supplementary file 1 — Supplementary Materials [file 41390_2023_2543_MOESM1_ESM.pdf]

Supplementary Material

Search string, Medline (OVID):

#	Searches
1	exp Heart Defects, Congenital/ or ((congenital* or hereditary or inborn) and ((heart* or cardiac* or coronary or septal* or aortopulmonary or aorticopulmonary or atrial or ventricular or intraventricular or biventricular) adj3 (defect* or disease* or malformation* or abnormal* or anomal* or "outflow tract obstruction"))).ti,ab. or (transpos* adj3 (arteries or artery or vessel*)).ti,ab. or ((pulmon* or aortic or subaortic or valve or mitral) adj1 stenosis).ti,ab. or ((aortic or aorta*) adj3 coarctation*).ti,ab. or ((interrupted or hypoplastic or anomal*) adj3 "aortic arch").ti,ab. or (heart adj3 hypoplas*).ti,ab. or ("single ventricle" adj3 "heart disease").ti,ab.
2	exp Infant/ or (newborn* or neonat* or postnat* or infant*).mp.
3	exp Cerebrovascular Circulation/ or exp Brain/bs or ((brain or cerebral or cerebrovascular) adj3 ("blood flow" or bloodflow or "blood supply" or "blood volume" or bloodvolume or circulation or hemodynamic* or haemodynamic* or perfusion)).ti,ab. or (Pulsatil* adj9 (cerebral or brain) adj1 arter*).ti,ab. or ((brain or cerebral or cerebrovascular) adj9 oxygen adj3 (saturat* or extract* or deliver* or consum*)).ti,ab. or ((brain or cerebral or cerebrovascular) adj12 (sao2 or so2 or OEF or CBF or CMRO2 or DO2 or cdo2 or VO2 or CVO2 or OEF or "t2*" or CBF)).ti,ab.
4	1 and 2 and 3
5	limit 4 to yr="1990 -Current"

Research protocol

Postnatal cerebral hemodynamics in infants with congenital heart disease: a scoping review

Background:

Children with severe congenital heart disease (CHD) are at risk for neurodevelopment impairment. It is known that brain growth and development in these children can be delayed and impaired. [1] Underlying causes and influencing factors are broadly discussed in recent literature.

Brain hemodynamics and metabolism had been discovered to be altered in fetuses and neonates with CHD. Common findings in cerebral ultrasound in fetuses with CHD are e.g. abnormal cerebroplacentar ratios (CPR) and cerebral pulsatility indices (PI)[2]; in neonates abnormal regional cerebral oxygenation saturations (rSO2) and cerebral blood flow rates (CBF) using near-infrared spectroscopy (NIRS) or magnetic resonance imaging (MRI)[3]. Brain development is dependent on optimal oxygen and nutrition supply, therefore the impact of these alterations on neurodevelopmental outcome are of high interest.

This research topic is challenged by substantial hemodynamic changes during brain development, with transition at birth and required surgical intervention after birth. Different imaging techniques are available to measure brain hemodynamics and metabolism including cerebral Doppler ultrasound and cerebral magnetic resonance imaging (MRI). A further challenge is the heterogeneous patient population consisting of a large variety of CHD types with different impact on cerebral perfusion.

Encountering the large amount of used technical methods, results and conclusions, the aim of this scoping review is to determine recent findings in brain hemodynamics for patients with severe CHD, and to identify their association with and influence on long-term neurodevelopment outcome.

Outcomes:

- Overview of methodological techniques used for measurement of cerebral perfusion
- Description of impaired neonatal brain development in different types of CHD
    - HLHS
    - TGA
    - Other single ventricle
    - Complex aortic arch anomaly

Presentation of findings:

- List of publications
- Development of findings from postnatal to infant life (until 12 months)
- Absolute or relative values, if possible in comparison with a healthy population
- no intraoperative measurements (after induction of anesthesia) or within 24h postop.
- Association of findings in CHD patients with neurodevelopmental outcome and mortality (cardiac outcome)

Methods:

A scoping review of literature that reports perfusion in human brain in CHD fetuses and neonates will be performed. Cochrane, EMBASE, Medline, Web of Science and Scopus will be searched.

Eligibility criteria

Studies to be included

Original, peer-reviewed research studies including population-based register studies, retrospective and prospective cohorts and cross-sectional studies will be included. Reviews, editorials or commentaries will be excluded, but screened for relevant studies. We have no restrictions to sample sizes.

Year of publication

Full papers will be included if published starting from 1990.

Language

No restrictions.

Participants

Included studies will report brain perfusion characteristics for patients with severe congenital heart diseases. Severe condition is defined by the necessity of surgical treatment within the first 3 months of life. Measurements of neonates and infants until one year of age will be included. For evaluation of impact on neurodevelopmental outcome, neurologic tests until 18 years of age will be included. Exclusion criteria are preterm infants or infants with associated genetic syndromes.

References

1. Mebius MJ, Kooi EMW, Hard CM, Bos AF. Brain Injury and Neurodevelopmental Outcome in Congenital Heart Disease: A Systematic Review. Pediatrics. 2017;140(1).

2. Donofrio MT, et al. Autoregulation of cerebral blood flow in fetuses with congenital heart disease: The brain sparing effect. Pediatric Cardiology. 2003;24(5):436-443.

3. Jain V, et al. Cerebral oxygen metabolism in neonates with congenital heart disease quantified by MRI and optics. Journal of Cerebral Blood Flow and Metabolism. 2014;34(3):380-388.
